# Clinical significance of increased cerebellar default-mode network connectivity in resting-state patients with drug-naive somatization disorder

**DOI:** 10.1097/MD.0000000000004043

**Published:** 2016-07-18

**Authors:** Houliang Wang, Wenbin Guo, Feng Liu, Jindong Chen, Renrong Wu, Zhikun Zhang, Miaoyu Yu, Lehua Li, Jingping Zhao

**Affiliations:** a Department of Psychiatry, Mental Health Institute of the Second Xiangya Hospital, Central South University, China National Clinical Research Center on Mental Disorders (Xiangya), China National Technology Institute on Mental Disorders, Hunan Technology Institute of Psychiatry, Hunan Key Laboratory of Psychiatry and Mental Health. Changsha, Hunan 410011, China; b Key Laboratory for NeuroInformation of Ministry of Education, School of Life Science and Technology, University of Electronic Science and Technology of China, Chengdu, Sichuan; c Mental Health Center, the First Affiliated Hospital, Guangxi Medical University, Nanning, Guangxi, China.

**Keywords:** cerebellum, default-mode network, functional connectivity, somatization disorder

## Abstract

The cerebellum has been proven to be connected to the brain network, as in the default-mode network (DMN), among healthy subjects and patients with psychiatric disorders. However, whether or not abnormal cerebellar DMN connectivity exists and what its clinical significance is among drug-naive patients with somatization disorder (SD) at rest remain unclear.

A total of 25 drug-naive patients with SD and 28 healthy controls were enrolled for a resting-state scan. The imaging data were analyzed using the seed-based functional connectivity (FC) method.

Compared with the controls, patients with SD showed increased left/right Crus I-left/right angular gyrus (AG) connectivity and Lobule IX-left superior medial prefrontal cortex (MPFC) connectivity. The FC values of the left/right Crus I-right AG connectivity of the patients were positively correlated with their scores in the somatization subscale of the symptom checklist-90 (Scl-90). A trend level of correlations was observed between the FC values of the left Crus I-left AG connectivity of the patients and their scores for the somatization subscale of Scl-90, as well as between the FC values of their Lobule IX-left superior MPFC connectivity and their scores for the Eysenck personality questionnaire (EPQ) extraversion.

Our findings show the increased cerebellar DMN connectivity in patients with SD and therefore highlight the importance of the DMN in the neurobiology of SD. Increased cerebellar DMN connectivities are also correlated with their somatization severity and personality, both of which bear clinical significance.

## Abstract 2

Supplemental Digital Content is available in the text

## 1 Introduction

Somatization disorder (SD) has a prevalence of 4% to 7% and is characterized by numerous medically unexplained somatic symptoms.^[1]^ The patients often contact their general physicians and repeatedly undergo medical examinations. However, the diagnosis of SD is difficult for general physicians to make, and this difficulty results in increasing health care utilization and medical cost.^[2]^ The availability of neuroimaging techniques may improve the understanding of the neurobiology of SD.^[3,4]^

The cerebellum has been recognized to have a role in cognition and emotion.^[5]^ For example, patients with olivopontocerebellar atrophy score higher in depression scales than healthy controls, and depression severity is weakly correlated with cognitive measurements.^[6]^ In healthy subjects, anger, sadness, and fear episodes bilaterally activate the midline cerebellum, whereas happy episodes activate the left cerebellum.^[7]^ However, the precise role of the cerebellum in cognition and emotion continues to be debated.

Anatomically, the cerebellum is linked to the cerebrum through 3 cerebellar peduncles. It has efferent and afferent fibers between the vermis, hypothalamus, and limbic system.^[5]^ Through these anatomical links, it is observed to have functional connectivity (FC) with brain networks, such as the Crus I and Lobule IX, as well as links to the default-mode network (DMN).^[8–12]^ The DMN encompasses a set of brain regions, including the medial prefrontal cortex (MPFC), posterior cingulate cortex (PCC), precuneus, and lateral parietal regions,^[13]^ and is involved in the self-referential process. Therefore, the cerebellum has a role in psychiatric illness. For example, abnormal cerebellar DMN connectivity has been found in first-episode and chronic schizophrenia,^[14–16]^ social anxiety disorder,^[17]^ and major depressive disorder.^[18]^ However, the alterations of cerebellar DMN connectivity in SD remain unclear.

Abnormality in the DMN has been shown in patients with SD via functional magnetic resonance imaging (fMRI). For example, regional alterations and abnormal FCs are observed in the bilateral superior MPFC, left precuneus,^[19]^ and left angular gyrus (AG).^[20,21]^ In addition, a positive correlation has been found between the FC values of the bilateral superior MPFC and the scores for the somatization subscale of the symptom checklist-90 (Scl-90).^[19]^ When confronted with emotional stimuli, patients with SD exhibit increased activity in the DMN, including the anteromedial thalamus (happy stimuli), posterior cingulate cortex (sad stimuli), and anterior ventral precuneus (happy and sad stimuli).^[22]^ Furthermore, increased FC strength in the right temporal gyrus^[23]^ and abnormal DMN homogeneity^[24]^ are observed in patients with SD. Interestingly, increased DMN homogeneity values in the bilateral precuneus are negatively correlated to the Eysenck personality questionnaire (EPQ) neuroticism scores in SD.^[24]^ These findings prove the abnormality of the DMN in SD, which bears clinical significance.

Although the alteration of the DMN in patients with SD is unequivocal, the alterations of the cerebellar DMN connectivity and its clinical significance in SD remain unclear. This study explores the cerebellar DMN connectivity in a group of drug-naive patients with SD. The first goal of this study is to investigate the altered FC pattern of the cerebellar DMN connectivity at rest. To our knowledge, no study has directly examined the correlation between the resting-state cerebellar DMN connectivity and clinical measurements (i.e., symptom severity, personality, and cognitive function) in patients with SD. Hence, our second goal is to examine the correlations between the abnormal cerebellar DMN connectivity and clinical measurements in SD. We hypothesize that patients with SD show increased cerebellar DMN connectivity. In addition, increased cerebellar DMN connectivity is expected to be correlated to clinical measurements in patients with SD.

## 2 Methods and materials

### 2.1 Participants

A total of 26 right-handed patients with SD were recruited from the Mental Health Center of the First Affiliated Hospital, Guangxi Medical University, China, and 30 right-handed healthy controls were recruited from the community. Handedness was determined by a multivariate approach using hand preference and hand performance measures.^[25]^ The age of the participants ranged from 18 to 50 years. The diagnosis of SD was made based on the criteria of the Structured Clinical Interview of the Diagnostic and Statistical Manual of Mental Disorders-IV (SCID), patient edition.^[26]^ The patients were at their first contact with the psychiatric department and were drug-naive to antidepressants. The controls were screened by SCID, non-patient edition.^[26]^ The participants shared the following exclusion criteria: severe medical or neurological diseases, mental retardation, any history of loss of consciousness, substance abuse, other psychiatric disorders, such as schizophrenia, anxiety disorders, bipolar disorders, or personality disorders, and any contraindications for MRI. Comorbidity with major depressive disorder was allowed given that patients with SD had a high rate of comorbidity with depression. However, the emergence of depressive symptoms should occur after the onset of somatic symptoms in the patients. In addition, potential controls with a first-degree relative with a history of neuropsychiatric disorders were excluded.

The participants were assessed with the following tests: the somatization subscale of Scl-90,^[27]^ Hamilton depression scale (HAMD, 17 items),^[28]^ and Hamilton anxiety scale (HAMA)^[29]^ to determine the symptom severity of their somatization, depression, and anxiety; EPQ^[30]^ to assess their personality dimensions; and Wisconsin Card Sorting Test (WCST)^[31]^ and Digit symbol-coding of Wechsler Adult Intelligence Scale (WAIS)^[32]^ to evaluate their cognitive function.

The study was approved by the local ethics committee of the First Affiliated Hospital, Guangxi Medical University. All the participants gave their written informed consent.

### 2.2 MRI acquisition

Functional MRI scans were acquired with a Siemens 3T scanner. The participants were asked to remain motionless and awake with their eyes closed. Soft earplugs and foam pads were used to reduce the scanner noise and head motion. The resting-state functional scans were obtained with a gradient-echo echo-planar imaging (EPI) sequence using the following parameters: repetition time/echo time = 2000/30 millisecond, 30 slices, 64 × 64 matrix, 90° flip angle, 24 cm FOV, 4 mm slice thickness, 0.4 mm gap, and 250 volumes (500 s).

### 2.3 Image preprocessing

The Data Processing Assistant for Resting-State fMRI^[33]^ was used to preprocess the scans. After slice timing and head motion correction, participants with more than 2 mm of maximal translation and 2° of maximal rotation were excluded. The scans were then normalized to the standard Montreal Neurological Institute (MNI) EPI space in SPM8 and resampled to 3 × 3 × 3 mm^3^. Subsequently, the scans were smoothed (with a 4 mm full width at half maximum Gaussian kernel), bandpass filtered (0.01–0.08 Hz), and linearly detrended. Several covariates, including the Friston-24 head motion parameters,^[34]^ the signal from a ventricular region of interest (ROI), and the signal from a region centered in the white matter, were removed. The global signal was not removed as indicated in a previous study.^[35]^

### 2.4 FC analysis

Three seed ROIs were employed in the right Crus I (MNI: 33, −76, −34), left Crus I (MNI: −33, −76, −34), and Lobule IX (MNI: 0, −55, −49). These seeds exhibited intrinsic connectivity with the DMN in patients with schizophrenia^[14,15]^ and healthy subjects.^[8,9]^ The seeds were defined as 6 mm radius spheres for FC analysis with the software REST.^[36]^ The Pearson correlation coefficients between each seed and other voxels of the entire brain were computed to create correlation maps that were *z*-transformed with Fisher *r*-to-*z* transformation.

For each seed and each group, one-sample *t* tests were used to detect voxels that showed significant correlations with the seed. The significance level was set at *P* < 0.005 and was corrected for multiple comparisons using Gaussian random field (GRF) theory (min *z* >2.807, cluster significance: *P* < 0.005, and a minimum cluster size of 20 voxels). After the normality of the data was assessed, a general linear model was established to compare group differences within the union mask of the results for the one-sample *t*-tests of the 2 groups. Age was used as a covariate to minimize the possible effect of this variable. The framewise displacement (FD) of each participant was calculated^[37]^ and used as covariate in the group comparisons. The HAMD and HAMA scores were also used as covariates to exclude the potential effects of depression and anxiety. The significance level was set at *P* < 0.005 (GRF corrected).

### 2.5 Correlation analysis

Once the clusters with abnormal FCs were determined in the patients, the mean *z* values of the brain clusters with abnormal FCs were extracted to examine the correlations between abnormal FCs and clinical variables of the patients. Multiple linear regressions were performed between these variables. The correlation results were Bonferroni corrected at *P* < 0.05.

## 3 Results

### 3.1 Sample characteristics

The images of 3 participants (1 patient and 2 controls) were excluded from the analysis due to excessive head motion. Six patients had comorbidity of major depressive disorder. As Table 1 shows, the 2 groups did not differ in terms of age, sex ratio, education level, EPQ extraversion/lie scores, Digit symbol-coding of WAIS, and WCST. As expected, the patients scored higher than the controls in HAMD, HAMA, somatization subscale of Scl-90, and EPQ Psychoticism/Neuroticism. The controls had higher scores in the FD values than the patients.

**Table 1:**
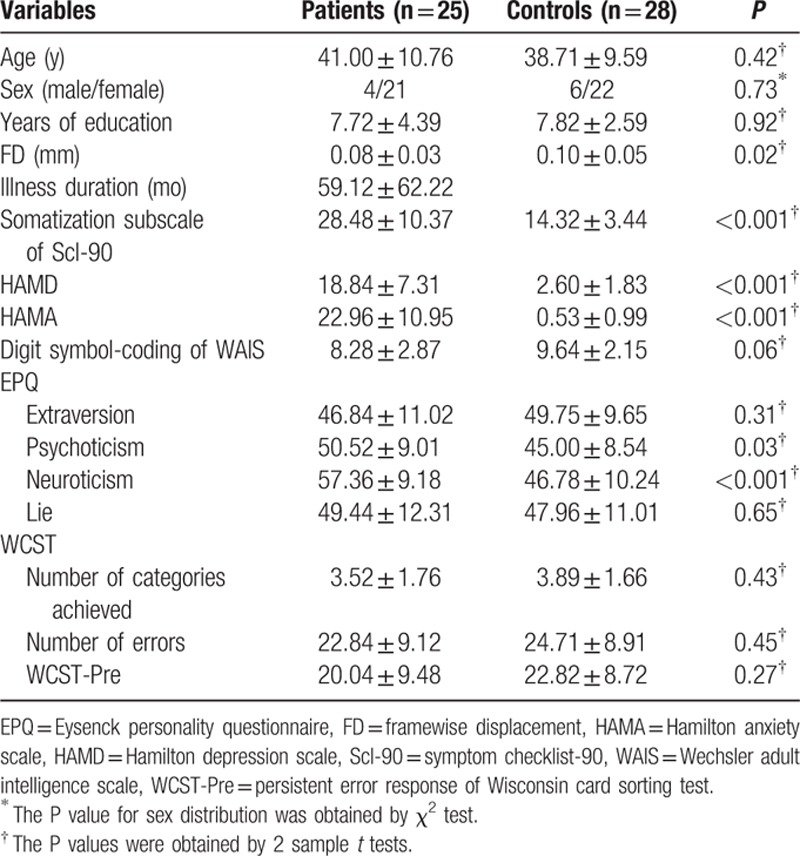
Sample characteristics of the participants.

### 3.2 FC patterns of the seed

The cerebellar seeds are exhibited in Figure S1. For each group, each cerebellar seed showed distributed FCs with the DMN using one-sample *t* tests (Figure S2). A union mask for each seed was created based on the results of one-sample *t* tests for the following analyses.

### 3.3 Seed-based FCs: Group differences

**Figure 1:**
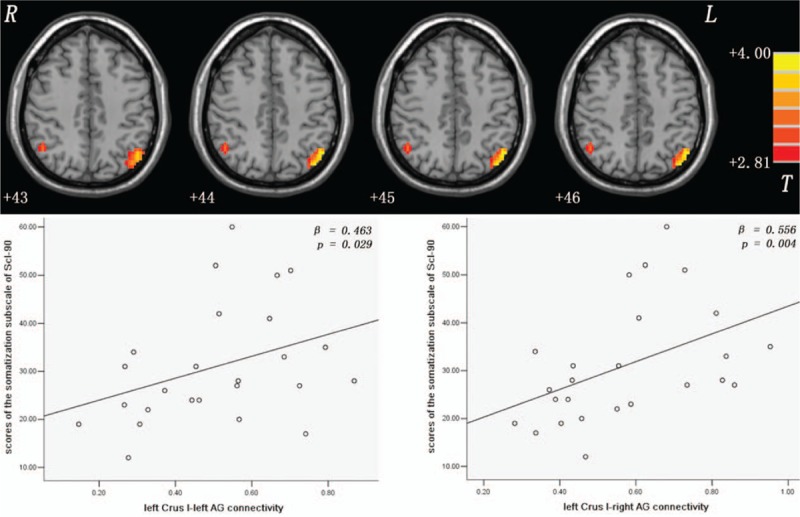
Increased left Crus I-left/right AG connectivity and their correlations with the scores for the somatization subscale of Scl-90 of patients with SD. The *P* values are uncorrected. The significance level is Bonferroni corrected at *P* < 0.05/5 = 0.01. Red denotes increased FC values. The color bar indicates the *t* values from the *t* tests of the 2 groups. AG = angular gyrus; FC = functional connectivity; Scl-90 = symptom checklist-90.

**Figure 2:**
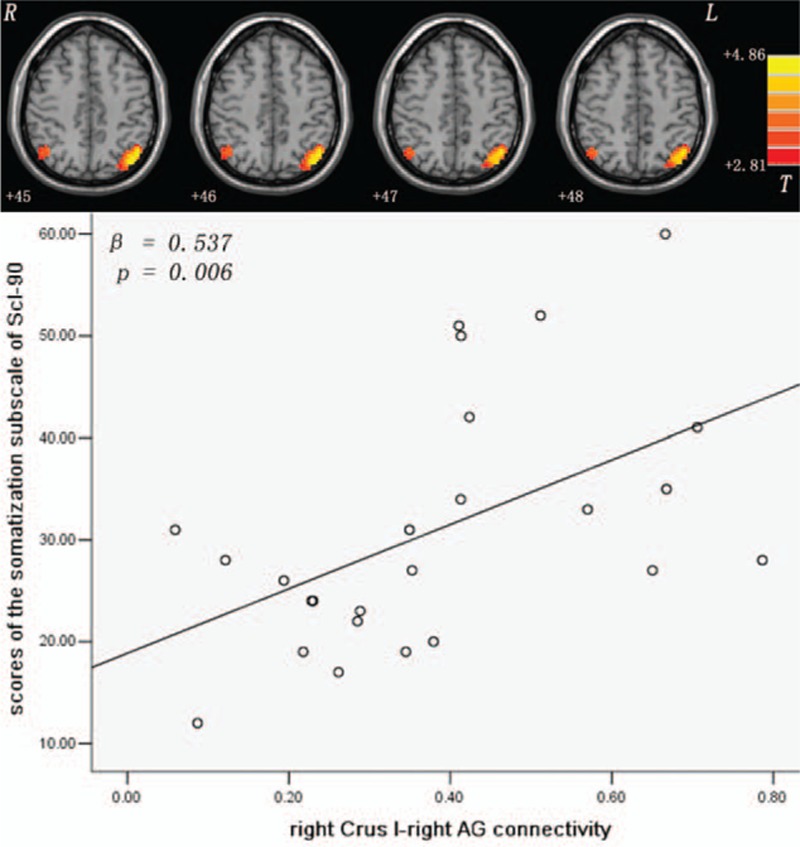
Increased right Crus I-right AG connectivity and its correlation with the scores for the somatization subscale of Scl-90 of patients with SD. The *P* values are uncorrected. The significance level is Bonferroni corrected at *P* < 0.05/5 = 0.01. Red denotes increased FC values. The color bar indicates the *t* values from the *t* tests of the 2 groups. AG = angular gyrus; FC = functional connectivity; Scl-90 = symptom checklist-90.

**Figure 3:**
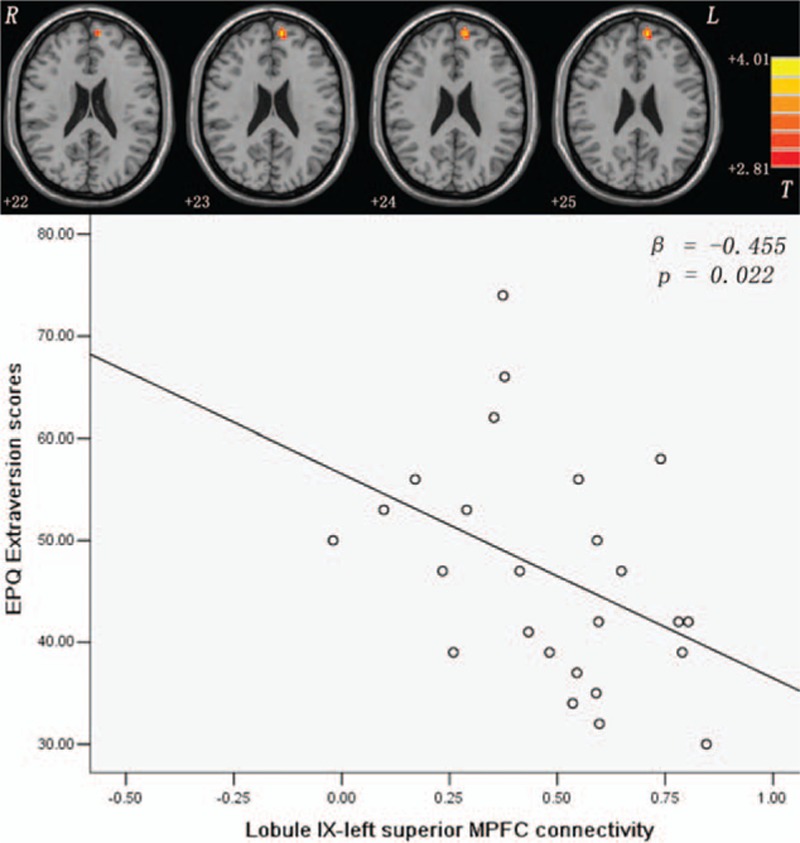
Increased lobule IX-left superior MPFC connectivity and its correlation with the EPQ extraversion scores of patients with SD. The *P* values are uncorrected. The significance level is Bonferroni corrected at *P* < 0.05/5 = 0.01. Red denotes increased FC values. The color bar indicates the *t* values from the *t* tests of the 2 groups. EPQ = Eysenck personality questionnaire; FC = functional connectivity; MPFC = medial prefrontal cortex.

Compared with the controls, the patients exhibited increased connectivity between the left/right Crus I and the left/right AG (Figs. 1 and 2 and Table 2), and between the Lobule IX and the left superior MPFC (Fig. 3 and Table 2). No significantly decreased connectivity was detected in the patients.

**Table 2:**
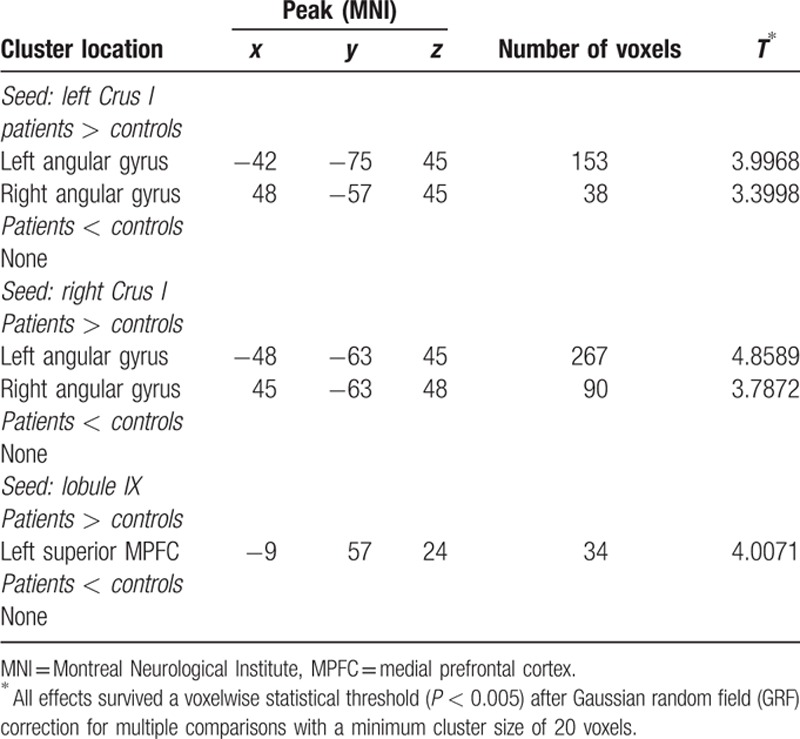
Brain regions with abnormal cerebellar connectivity between the patients and the controls.

### 3.4 Correlations between abnormal FCs and clinical variables

In the patients, the mean *z* values of the left/right Crus I-right AG connectivity were positively correlated with the scores of the somatization subscale of Scl-90 (left Crus I: *β* = 0.556, *P* = 0.004, right Crus I: *β* = 0.537, *P* = 0.006, Bonferroni corrected at *P* < 0.05/5 = 0.01; Figs. 1 and 2). A trend level of correlations existed between the mean *z* values of the left Crus I-left AG connectivity and the scores for the somatization subscale of Scl-90 (*β* = 0.463, *P* = 0.029, uncorrected; Fig. 1), and between the mean *z* values of the Lobule IX-left superior MPFC connectivity and the EPQ extraversion scores (*β* = -0.455, *P* = 0.022, uncorrected; Fig. 3) of the patients. No correlations were found between the abnormal FC values and cognitive variables (WCST and Digit symbol-coding of WAIS) of the patients.

## 4 Discussion

To our knowledge, this study is the first to examine cerebellar FC patterns with the DMN among drug-naive patients with SD. The findings reveal that patients with SD have increased cerebellar DMN connectivity, including the Crus I-AG connectivity and the Lobule IX-left superior MPFC connectivity. Another important finding is that the Crus I-right AG connectivity is positively correlated with the scores for the somatization subscale of Scl-90, whereas the Lobule IX-left superior MPFC connectivity has a trend level of negative correlation with the EPQ extraversion scores, indicating that the present findings bear clinical significance.

Two interesting findings emerge from this study. First, increased cerebellar DMN connectivity is observed among drug-naive patients with SD. Increased FC is often conceived as compensatory reallocation or dedifferentiation.^[23,38–41]^ Inflammation may modulate the compensatory process.^[42]^ In the early course of SD, proinflammatory cytokines (i.e., tumor necrosis factor and interleukin-6) activate the astrocytes and show hyperfunction (increased blood flow and metabolism).^[43]^ Regional hyperfunction can contribute to the increased FC and activity in this region. Moreover, increased connectivity in the DMN has been found in early-course patients with SD.^[23]^ Therefore, it is no wonder that patients with SD exhibit increased cerebellar DMN connectivity in this study.

Another interesting finding is that increased cerebellar DMN connectivity is correlated with clinical variables. First of all, the left/right Crus I-right AG connectivity has a positive correlation with the scores of the somatization subscale of Scl-90, suggesting that patients with a higher left/right Crus I-right AG connectivity experience an increased severity of somatic symptoms. As a key brain region of the DMN, AG is involved in number processing, language, memory retrieval, and attention.^[13]^ AG also has a crucial role in conceptual metaphors and cross-modal abstractions.^[44]^ Increased regional activity in the AG with a positive correlation with the EPQ Neuroticism scores has been reported in patients with SD,^[20]^ suggesting that patients with higher EPQ Neuroticism scores would exhibit higher regional activity in the AG. Individuals with higher EPQ Neuroticism scores are apt to have emotional disturbances.^[30]^ It is therefore explained why patients with SD suffer a high rate of comorbidity with major depressive disorder and/or anxiety disorders. The functional alterations of the AG may lead to awareness, emotional, and behavioral abnormality of the patients.^[45]^ For example, when AG is activated, a female subject perceives a phantom existence behind her, indicating that the hyperfunctioning of the AG may easily cause uncomfortable feelings in the perceived position of the body instead of its actual location.^[46]^ This phenomenon may explain why somatic symptoms are medically unexplained among patients with SD. It may also explain the present correlations among patients with a higher left/right Crus I-right AG connectivity experiencing an increased number of somatic symptoms.

Furthermore, a trend level of negative correlation is observed between the Lobule IX-left superior MPFC connectivity and the EPQ extraversion scores among patients with SD. MPFC, a key node of the DMN, has wide links to the affective limbic regions, such as the hippocampus, amygdala, and hypothalamus. It has a crucial role in self-referential processing, emotional regulation, and extinction memory.^[47]^ Increased Lobule IX-left superior MPFC connectivity is found among patients with SD in this study, which is consistent with its crucial role in emotion and cognition. MPFC may be connected to the cerebellum through the frontal-thalamic-cerebellar circuit.^[5]^ The trend level of negative correlation suggests that introverted patients have a high Lobule IX-left superior MPFC connectivity. Introversion is characterized by quietness, low positive affect, and the need for internal stimuli. Moreover, a positive correlation was reported between increased regional activity of the bilateral superior MPFC and somatization subscale scores of the Scl-90 in patients with SD,^[19]^ suggesting that regional activity of the superior MPFC can be used as an index for the severity of somatic symptom in patients with SD. In line with this finding^[19]^ and the personality characteristic of focusing on internal stimuli, introverts may pay much attention to their internal body discomfort and be apt to have a high rate of suffering from SD. Therefore, the present results highlight the importance of personality in the neurobiology of SD.

This study has several limitations, aside from its relatively small sample size. First, it is difficult for us to disentangle SD from major depressive disorder and/or anxiety disorder, and patients with comorbid major depressive disorder were included in this study. Although we applied the HAMD and HAMA scores as covariates to reduce the possibility that the depression and/or anxiety in patients with SD contributed to the present findings, we may not completely eliminate the effects caused by the depression and anxiety. Second, this study is cross-sectional. A follow-up study is needed to elucidate the alterations of the cerebellar DMN connectivity after treatment. Third, this study focuses on the DMN and enhances the specificity of the findings on the DMN. For the same reason, other meaningful results from other networks, such as salience network and executive control network, may be neglected. Finally, the MRI data are acquired at resting state. Hence, the increased cerebellar DMN connectivity reported here is associated with somatic pathophysiology in general, and not with a specific pattern of thought or activity.

Despite its limitations, this study reveals increased cerebellar DMN connectivity among patients with SD and therefore highlights the importance of the DMN in the neurobiology of SD. Increased cerebellar DMN connectivities are also correlated with somatization severity and personality, suggesting that such connectivities bear clinical significance.

## Supplementary Material

Supplemental Digital Content
