# Supplementary material for: Clinical significance of increased cerebellar default-mode network connectivity in resting-state patients with drug-naive somatization disorder
Source: Medicine (Baltimore). 2016 Jul 18;95(28):e4043. doi: 10.1097/MD.0000000000004043 (PMC4956784; doi:10.1097/MD.0000000000004043)

Figure S1. Six mm-radius sphere cerebellar seeds for the FC analysis. FC = functional connectivity.


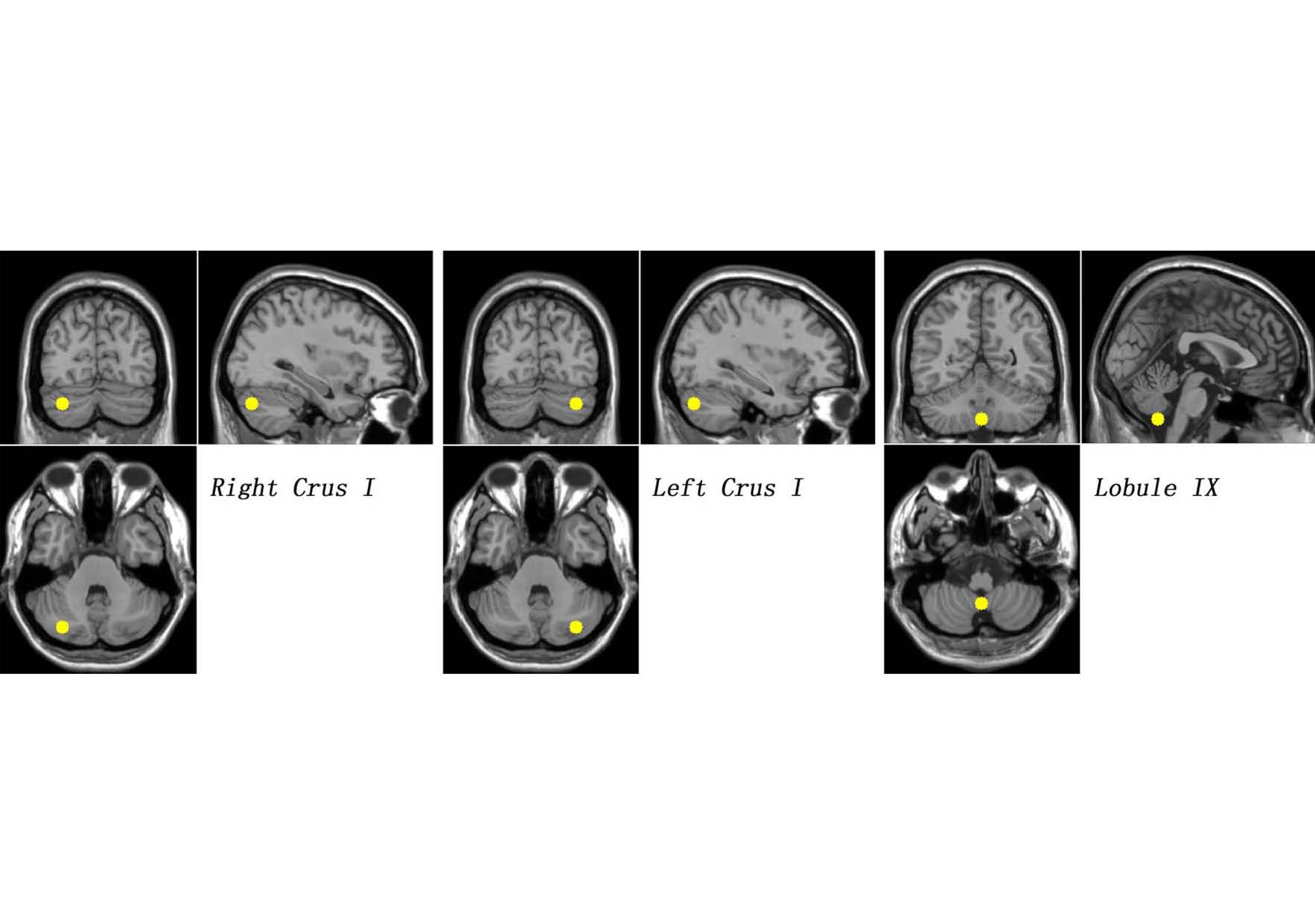


Figure S2. Cerebellar seeds have FCs with the default-mode network among both the patients with SD and the controls. Red denotes increased FC values. The color bar indicates the *t* values from the one-sample *t*-tests. FC = functional connectivity.


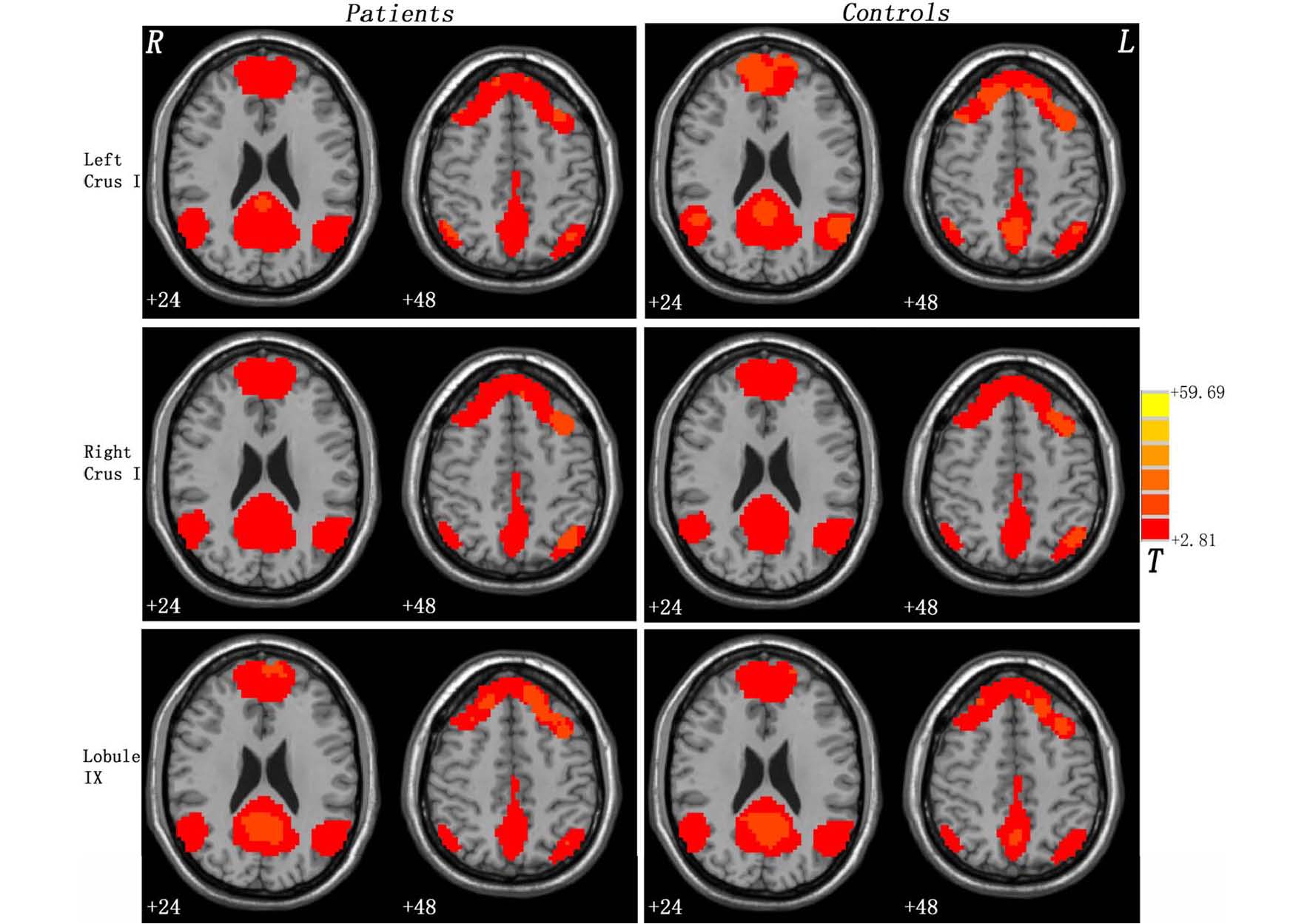

Supplement: Supplemental Digital Content [file medi-95-e4043-s001.doc]
